# A comparison of posterior lumbar interbody fusion and transforaminal lumbar interbody fusion: a literature review and meta-analysis

**DOI:** 10.1186/1471-2474-15-367

**Published:** 2014-11-05

**Authors:** Qunhu Zhang, Zhen Yuan, Min Zhou, Huan Liu, Yong Xu, Yongxin Ren

**Affiliations:** Department of Orthopedics, The First Affiliated Hospital of Nanjing Medical University, 300 Guangzhou Road, Nanjing, 210029 Jiangsu People’s Republic of China

**Keywords:** Posterior lumbar interbody fusion, Transforaminal lumbar interbody fusion, Meta-analysis, Lumbar fusion

## Abstract

**Background:**

We compared the perioperative results and complications associated with PLIF and TLIF, and collected evidence for choosing the better fusion method.

**Methods:**

A literature survey of the MEDLINE and EMBASE databases identified 7 comparative observational studies that met our inclusion criteria. Checklists by Cowley were used to evaluate the risk of bias of the included studies. A database including patient demographic information, perioperative results, and complications was established. The summary odds ratio and weighed mean difference with 95% confidence interval were calculated with a random-effects model.

**Results:**

We found that PLIF had a higher complication rate (P <0.00001), and TLIF reduced the rate of durotomy (P = 0.01). No statistical difference was found between the two groups with regard to clinical satisfaction (P = 0.54), blood loss (P = 0.14), vertebral root injury (P = 0.08), graft malposition (P = 0.06), infection (P = 0.36), or rate of radiographic fusion (P = 0.27). The evidence indicated that PLIF required longer operative time (P = 0.03).

**Conclusions:**

The evidence indicated that TLIF could reduce the complication rate and durotomy. Neither TLIP nor PLIF was found superior in terms of clinical satisfaction or radiographic fusion rate. PLIF might result in longer time in surgery.

**Electronic supplementary material:**

The online version of this article (doi:10.1186/1471-2474-15-367) contains supplementary material, which is available to authorized users.

## Background

The optimal treatment for degenerative lumbar diseases remains controversial [1, 2]. The currently recommended surgical procedures are the anterior lumbar interbody fusion, the posterior lumbar interbody fusion (PLIF), and the transforaminal lumbar interbody fusion (TLIF) [3–7]. The PLIF and TLIF are the more commonly implemented [8–12].

PLIF was first described by Cloward in 1940 and became commonly used after modifications were proposed by Lin [13, 14]. PLIF can only be performed through the posterior approach, and it enables a stable three-column fixation with 360° fusion and anterior support [15–17]. In 1982, Harms and Rolinger first described the TLIF technique for creating a circumferential fusion via a single posterolateral approach [18]. As reported by Audat et al., in 1998 Harms and Rolinger reported the treatment outcomes of 191 patients who received TLIF between 1993 and 1996 [19, 20]. This procedure involves the placement of pedicle screws and an interbody spacer via a single posterolateral route.

Many studies have compared methods for lumbar interbody fusion with regard to clinical results and fusion rates [20–22]. However, the inconsistent results of these studies do not provide sufficient evidence to determine which is the optimal fusion technique. The present study is a meta-analysis, conducted to provide cumulative effect estimates of clinical outcomes and to determine which surgical technique is more beneficial.

## Methods

### Search strategy and inclusion criteria

A survey was conducted of literature published until June 2013 using the MEDLINE and EMBASE databases. All fields were screened using the key terms “posterior lumbar interbody fusion” or “PLIF” combined with “transforaminal lumbar interbody fusion” or “TLIF”. Pertinent articles in reference lists were also inspected.

Studies were included in this meta-analysis if they met the following criteria: 1) the study design was comparative (i.e., PLIF compared to TLIF); 2) the study population consisted of adult patients suffering from degenerative lumbar diseases (disc herniation, spinal stenosis, or spondylolisthesis); 3) the study reported at least one desirable outcome regarding perioperative results (e.g., operative time, blood loss), complications, pain or disability improvement, or fusion rate; 4) the patients were followed up for at least 6 months after surgery; and 5) each group comprised at least 10 patients. Excluded from the present meta-analysis were case reports, reviews, biomechanical and cadaveric studies, and repeated studies.

### Data extraction and quality assessment

The following information was extracted from each publication: 1) the first author’s last name, study year, country and study design; 2) basic study characteristics including the number of enrolled patients, age, and gender proportion; 3) perioperative results such as operative duration, blood loss, and hospitalization; 4) complication types and rates; and 5) fusion rate. Both intraoperative and postoperative complications were extracted. Complication types were defined as in previous published reviews [18].

The quality of the included studies was evaluated using the Cowley criteria. A Cowley score ≥9 out of a possible 17 was considered high methodological quality [23, 24].

### Meta-analysis

The analysis was conducted with the statistical software Review Manager Version 5.2 (Cochrane Collaboration) using a random effects model. Continuous outcomes were calculated by the weighted mean difference (WMD) and 95% confidence interval (CI). Dichotomous variables were summarized using the odds ratio (OR) and 95% CI. Heterogeneity was evaluated using I2 statistics. I2 values of <25%, 25-50%, 50-75%, and >75% were considered to indicate no, low, moderate, and high heterogeneity, respectively. Funnel plots were employed to assess the possibility of publication bias.

## Results

### Literature surve

**Figure 1:**
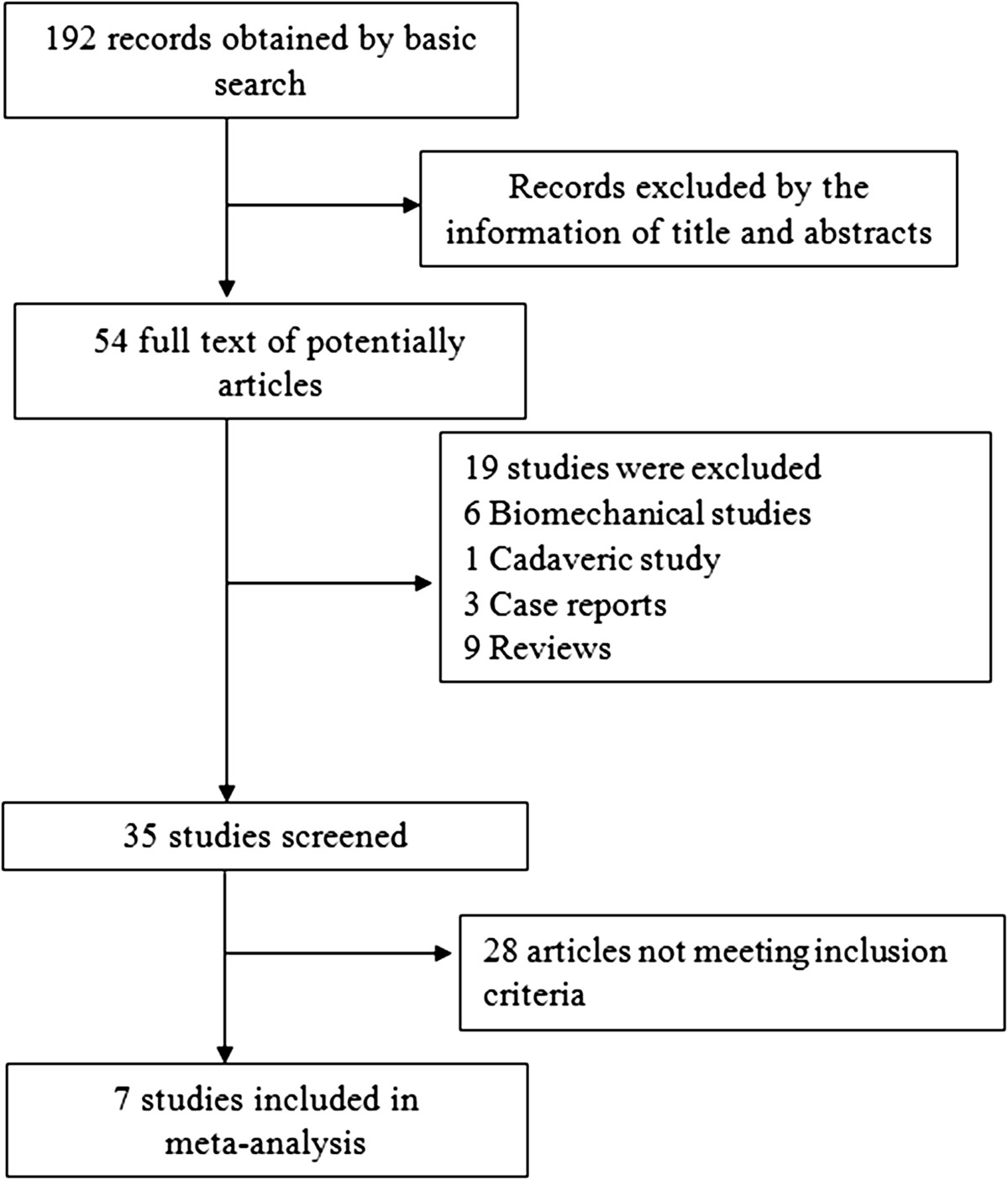
**Flow diagram of the selection process.**

Seven non-randomized comparative studies were identified (Figure 1). The basic strategy yielded 192 records. Fifty-four full texts were screened by titles and abstracts. Nineteen case reports, reviews, and biomechanical and cadaveric studies were excluded. No eligible studies were found in the search of the references of the retrieved articles. Finally, the outcomes of 647 patients were included in the meta-analysis.

### Study characteristics

Four studies consisted of patients who had received a diagnosis of degenerative disc disease [19–22] and in four studies lumbar instability and spondylolisthesis had been diagnosed (Table 1) [19, 22, 25, 26]. One study comprised patients with recurrent lumbar disc protrusion [27].

**Table 1:** Characteristics and evaluation of included studies*

	Year	Country of origin	PLIF/TLIF				Evaluation
			Patients, n	Mean age, y	Male, %	Mean follow up, months	
Humphreys et al. [19]	2001	USA	34/40	40.00/41.00	64.71/50.00	13.0/13.0	10
Jae-Sung Park et al. [22]	2005	Korea	99/29	54.00/57.00	43.43/34.48	20.0/10.4	12
Yan et al. [25]	2008	China	60/60	63.60/64.50	55.00/53.33	23.0/23.0	14
Zhuo et al. [27]	2009	China	22/18	41.00/43.00	63.64/72.22	20.0/20.0	13
Mehta et al. [21]	2011	USA	76/43	48.56/48.12	44.74/37.21	24.0/24.0	11
Audat et al. [20]	2012	Jordan	27/37	50.60/45.80	22.22/37.84	36.0/36.0	13
Sakeb et al. [26]	2013	Bangladesh	52/50	46.73/49.04	21.15/28.00	9.0/9.0	14

*All studies were retrospective and comparative.

The included articles were scored in accordance with the Cowley criteria (Table 1). The Cowley scores of the 7 comparative studies ranged from 10 to 14 out of a possible 17. Therefore, the included studies were considered of high methodological quality.

### Meta-analysis results

#### Complications

**Figure 2:**
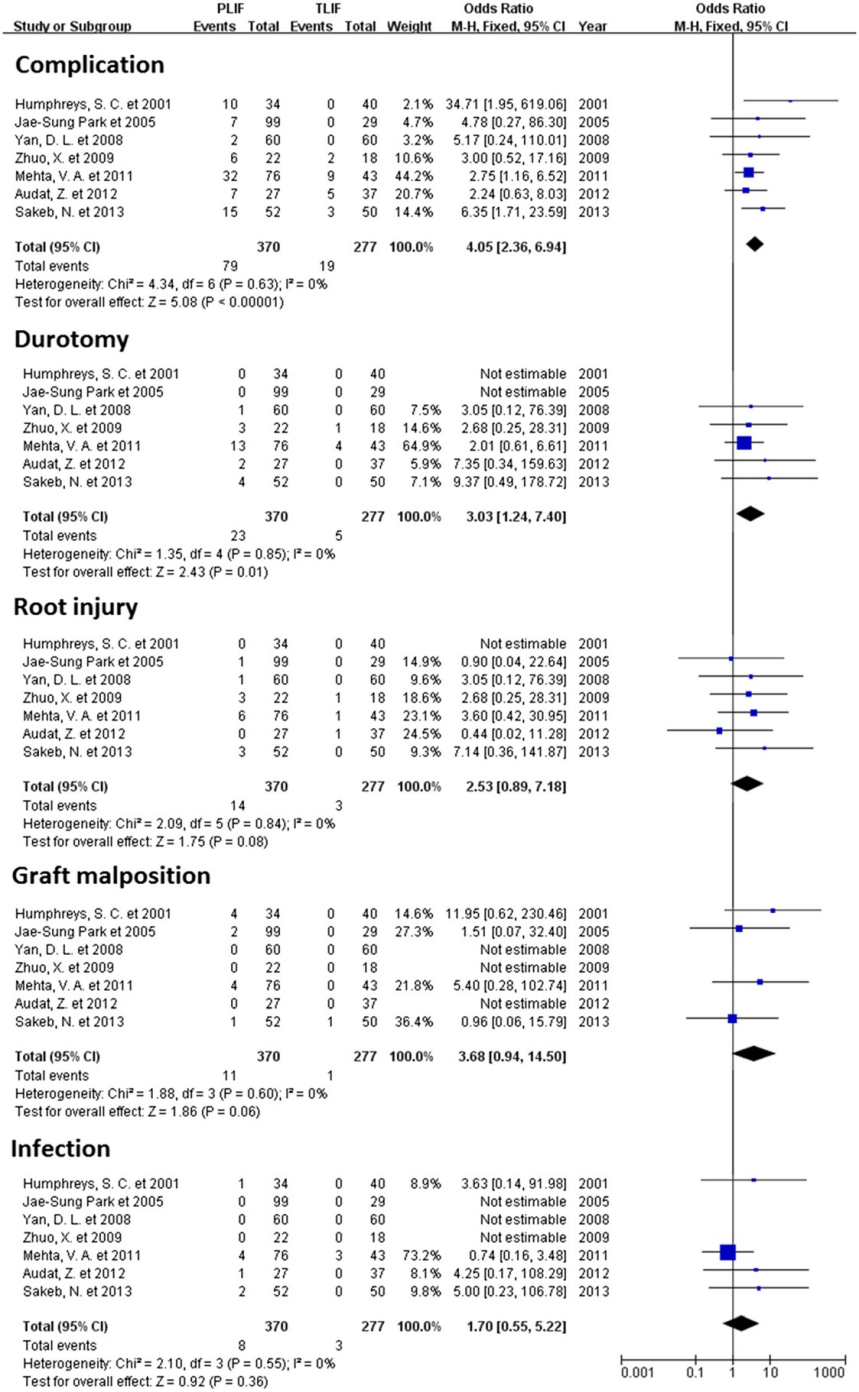
**Forest plot illustrating complication rate of PLIF and TLIF.**

All of the 7 studies reported complications associated with surgery [19–22, 25–27]. Durotomy, root injury, infection, cerebrospinal fluid leakage, and implant migration were observed [28–30]. The overall complication rate was significantly higher in the PLIF group than the TLIF (OR 4.05, 95% CI: 2.36 to 6.94, P <0.00001). There was no evidence for significant heterogeneity (I^2^ = 0%, P = 0.63; Figure 2).

Durotomy was reported in 5 studies [21, 22, 25–27], and the overall difference in durotomy rates was statistically significant (OR 3.03, 95% CI: 1.24 to 7.40, P = 0.01). Statistical heterogeneity was not detected among the studies with regard to durotomy (I^2^ = 0%, P = 0.85; Figure 2).

The rates of root injury (OR 2.53, 95% CI: 0.89 to 7.18, P = 0.08), graft (pedicle screw, cage, and bone graft) malposition (OR 3.68, 95% CI: 0.94 to 14.50, P = 0.06) and infection (OR 1.70, 95% CI: 0.55 to 5.22, P = 0.36) were similar between the TLIF and PLIF groups. There was no evidence of significant heterogeneity with regard to complications (I^2^ = 0%, P >0.1; Figure 2). One study reported cerebrospinal fluid leakage but the difference was not statistically significant between the two groups [21].

### Clinical satisfaction

**Figure 3:**
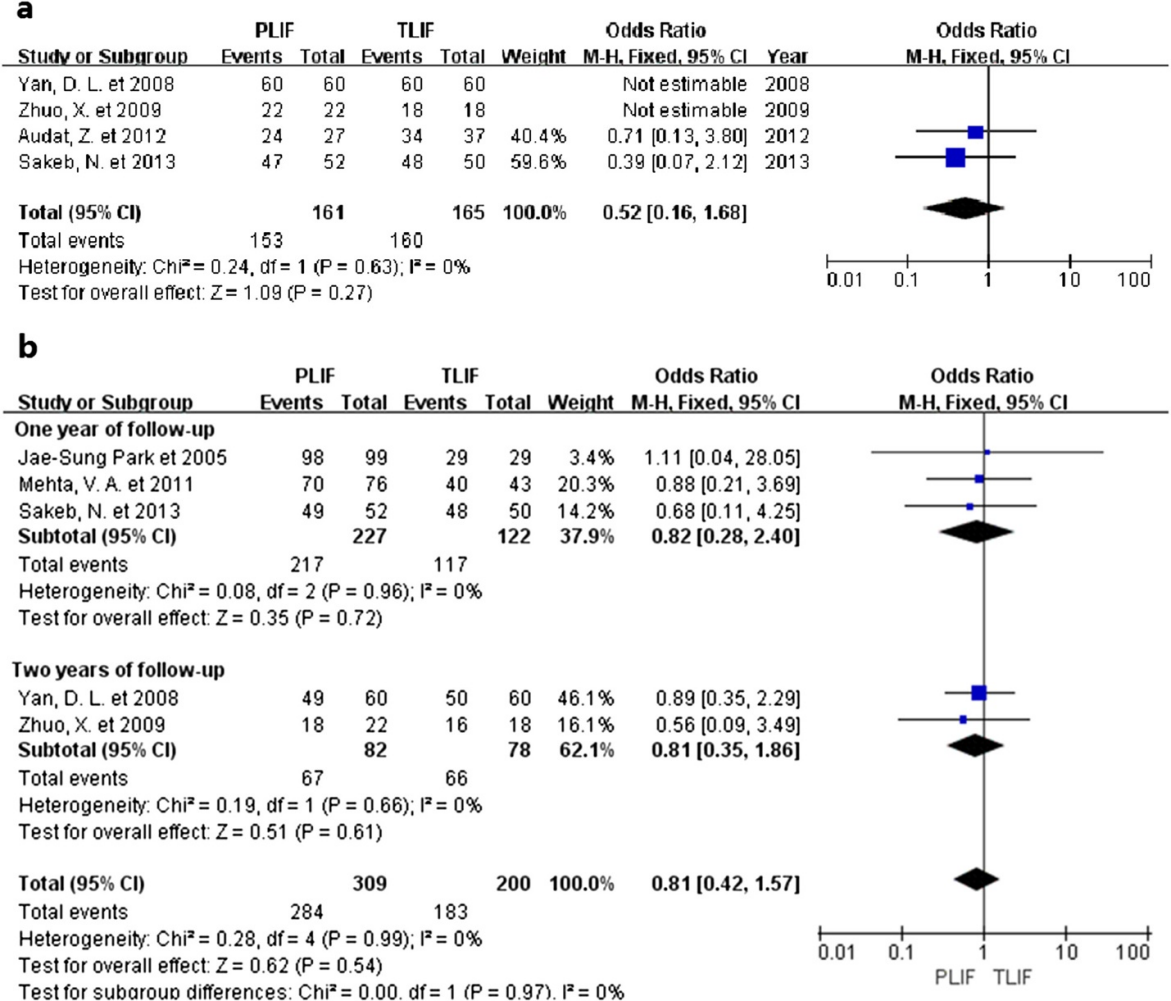
**Forest plot illustrating radiographic fusion (a) and satisfaction (b) of PLIF and TLIF.**

Five studies reported evaluations of satisfaction made by patients, or clinical satisfaction was assessed based on the Oswestry disability index (ODI) or Japanese Orthopedic Association (JOA) scores. No statistical difference was found between the two groups (OR 0.81, 95% CI: 0.42 to 1.57, P = 0.54). There was no evidence for significant heterogeneity (I^2^ = 0%, P = 0.99; Figure 3). The evidence from one-year follow-up studies showed that there was no statistically significant difference between the two procedures (OR 0.82, 95% CI: 0.28 to 2.40, P = 0.72) [21, 22, 26]. The follow-up period of two studies was two years [25, 27], and no statistical difference was found (OR 0.81, 95% CI: 0.35 to 1.86, P = 0.61). Subgroup analysis showed similar trends in the one-year and two-year follow-up periods.

### Fusion rate

Data regarding fusion rates were available in four studies and none of them reported statistically significant differences [22, 25–27]. Overall, the fusion rate was similar for both groups (OR 0.52, 95% CI: 0.16 to 1.68, P = 0.27). Chi-squared tests indicated no statistical evidence of heterogeneity (I^2^ = 0%, P = 0.63; Figure 3).

### Operative time

**Figure 4:**
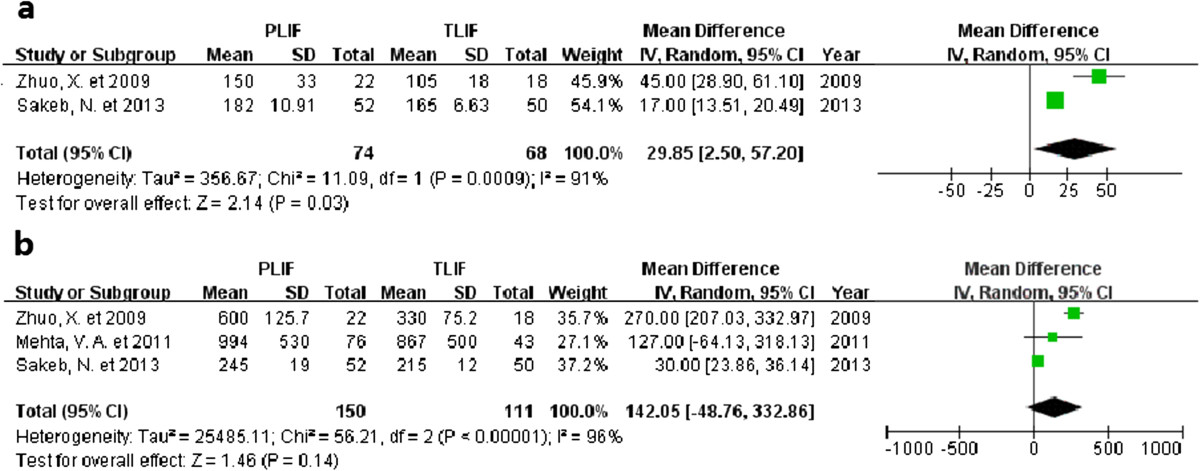
**Forest plot illustrating operative time (a) and blood loss (b) of PLIF and TLIF.**

Operative time was recorded in four studies [19, 22, 26, 27] and two studies reported statistically significant differences showing that PLIF requires more time [26, 27]. Two of them provided the mean and P values [19, 22]. Two studies reported adequate mean and standard deviation data [26, 27]. The WMD for operative time was 29.85 minutes longer for the PLIF group than the TLIF group (95% CI: 2.50 to 57.20), P = 0.03). High heterogeneity existed among the studies (I^2^ = 91%, P = 0.0009; Figure 4).

### Blood loss

Details regarding intraoperative blood loss were available in four studies [19, 21, 26, 27]. Three studies provided mean and standard deviation [21, 26, 27]; one study reported mean and P-value. Two studies showed statistically significant differences [26, 27]. Overall, the WMD was equivalent for both the PLIF and TLIF groups (WMD = 142.05, 95% CI: −48.76 to 332.86, P = 0.14). There was obvious evidence for statistically significant heterogeneity (I^2^ = 96%, P <0.00001; Figure 4).

### Sensitivity analysis

Sensitivity analysis was conducted by reanalyzing the data after sequential omission of individual studies. Pooled results did not yield any significant difference after omitting the data of any single study.

## Discussion

The evidence of this updated meta-analysis indicated that PLIF results in a higher complication rate than does TLIF, based on 7 high methodological quality studies of epidemiological data. PLIF required longer operative time than TLIF, while high heterogeneity existed among the studies. No significant difference was found between the two procedures regarding blood loss, clinical satisfaction, or fusion rate.

PLIF was associated with a significantly higher complication rate. However, since the types of complications or their definitions were inconsistent among the studies, pooling the complication data might lead to bias. The main complication types included durotomy, root injury, graft (pedicle screw, cage, and bone graft) malposition, and infection [28–30]. Compared with TLIF, the incidence of durotomy was higher in PLIF. A trend in increased risk of root injury and graft malposition was also observed for PLIF, although the difference was not statistically significant. Because the approach in the TLIF technique is lateral to the vertebral foramen, there is less retraction of the dura or conus medullaris, and greater protection of the spinous processes that can affect postoperative spinal stability [31–36]. This leads to a lower incidence of durotomy and root injury in TLIF groups than in the PLIF. Our present meta-analysis supports this theoretical assumption, and may explain the lower incidence of graft malposition in TLIF. Because TLIF preserves the posterior compartment more effectively than PLIF does, transitional syndrome or screw fracture is less likely to occur.

The PLIF procedure required longer operative time. However, data among the studies were highly heterogeneous. A precise pooled mean difference could not be calculated because the number of studies providing the mean and standard deviation were insufficient. The broad dissection performed in PLIF might increase the operative time; but we still need more clinical data to support this conclusion. Over the past few years, modifications and refinements of surgical techniques have continually been made to achieve better outcomes [37–40]. Such modifications include minimization of neural retraction and avoidance of broad dissection of the paraspinal musculature. Efforts were also made to reduce complication rates and to develop minimally invasive spine surgery, which results in less blood loss [34, 35, 41]. Postoperative hospitalization was also reported [21, 22, 26, 27], and the P-value of these studies indicated no statistically significant difference.

For spinal surgeries, a better surgical technique should induce fewer complications as well as less blood loss. It is consistent with Takahashi et al. findings in 2011 [42]. TLIF is a satisfactory choice. Minimal invasive spine surgeries are expected to be adopted more and more widely in future clinical trials [43–47].

As with other systematic reviews, there were limitations to this study. Firstly, there were no high-quality randomized controlled trials included, and these are increasingly important in the evaluation of surgical treatments [48–50]. Furthermore, the sample size in some subgroup analyses was quite small. When continuous outcomes were pooled, statistical heterogeneity was evident; this might be explained by differences in study design and quality, patients’ characteristics, and the diverse technical specifications. A further limitation is that clinical outcome data was sometimes incomplete. Finally, by its nature meta-analysis is just a statistical test that is subject to many methodological restrictions and is not able to control all relevant factors. Despite the above weaknesses, the present meta-analysis still has academic value.

## Conclusions

TLIF was shown to result in a lower complication rate, and PLIF was associated with an increased risk of durotomy. In addition, low-quality evidence showed that the PLIF required longer operative time than the TLIF. Regarding blood loss and fusion rates, there was no significant difference between the two fusion procedures.

## Authors’ information

Qunhu Zhang and Zhen Yuan: The first two authors should be regarded as joint First Authors.

## Authors’ original submitted files for images

Below are the links to the authors’ original submitted files for images. Authors’ original file for figure 1 Authors’ original file for figure 2 Authors’ original file for figure 3 Authors’ original file for figure 4 Authors’ original file for figure 5
