# Supplementary figures and images for: A comparison of posterior lumbar interbody fusion and transforaminal lumbar interbody fusion: a literature review and meta-analysis
Source: BMC Musculoskelet Disord. 2014 Nov 5;15:367. doi: 10.1186/1471-2474-15-367 (PMC4232693; doi:10.1186/1471-2474-15-367)

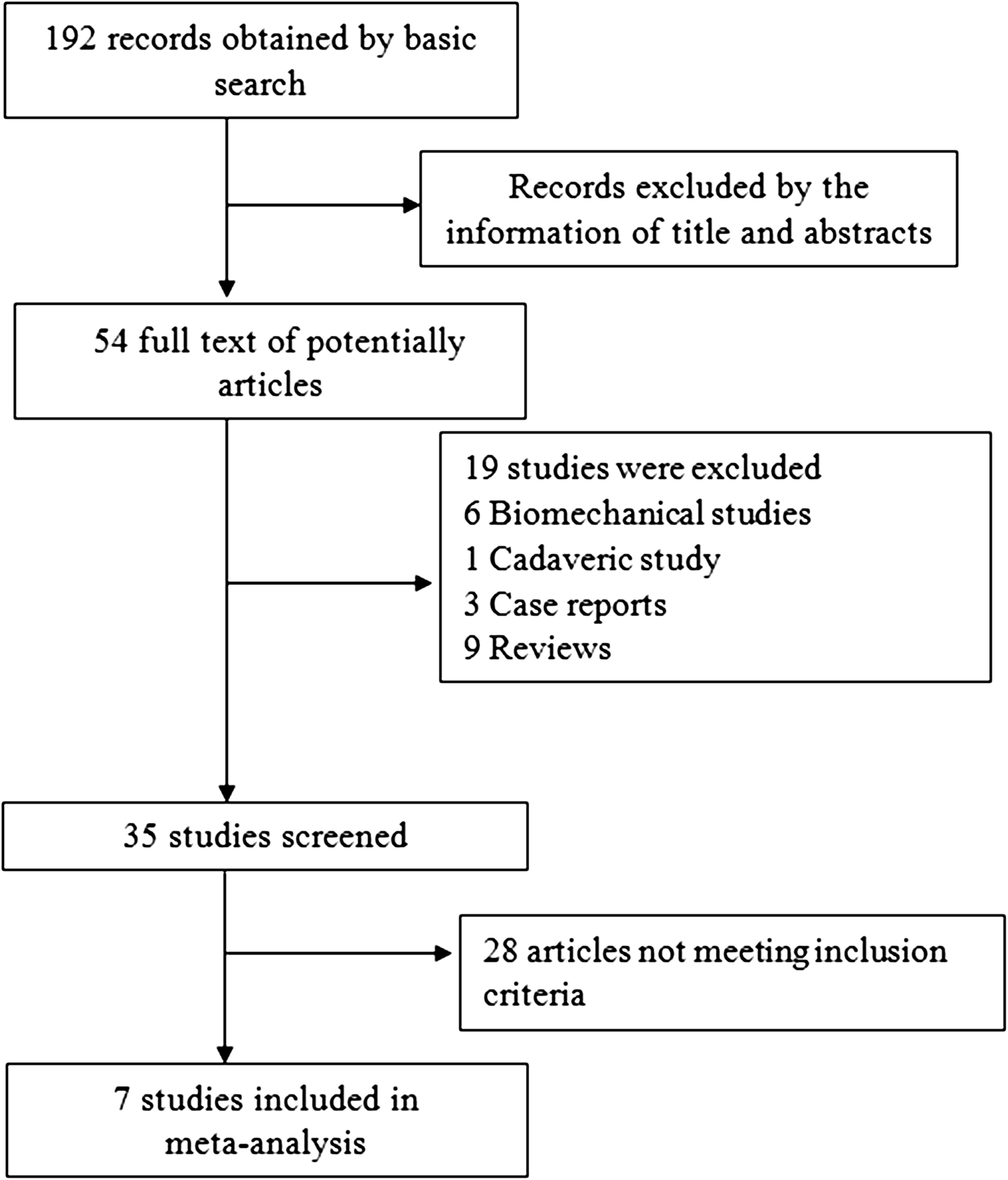

Supplement: Supplementary file 1 — Authors’ original file for figure 1 [file 12891_2014_2303_MOESM1_ESM.tif]

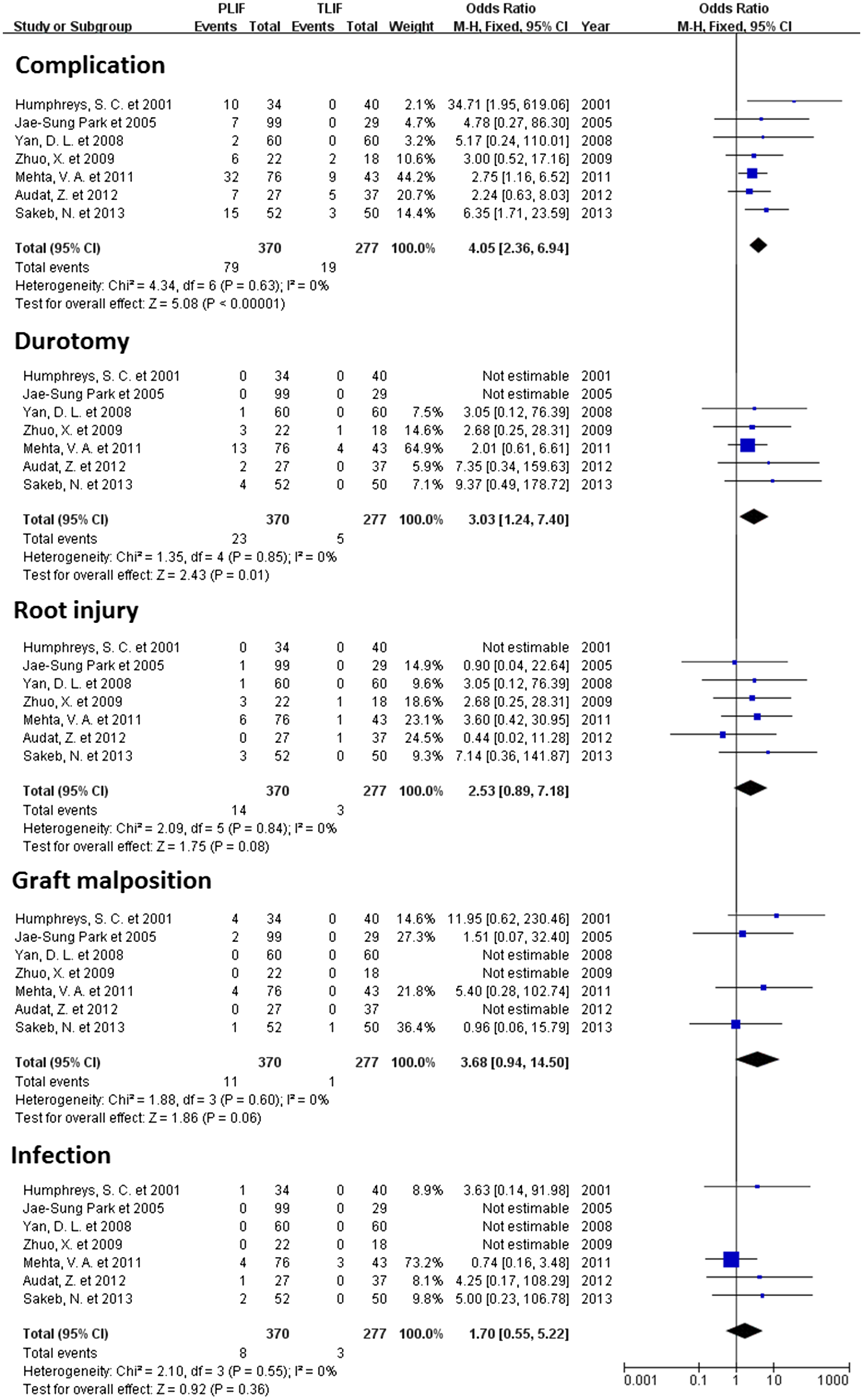

Supplement: Supplementary file 2 — Authors’ original file for figure 2 [file 12891_2014_2303_MOESM2_ESM.tif]

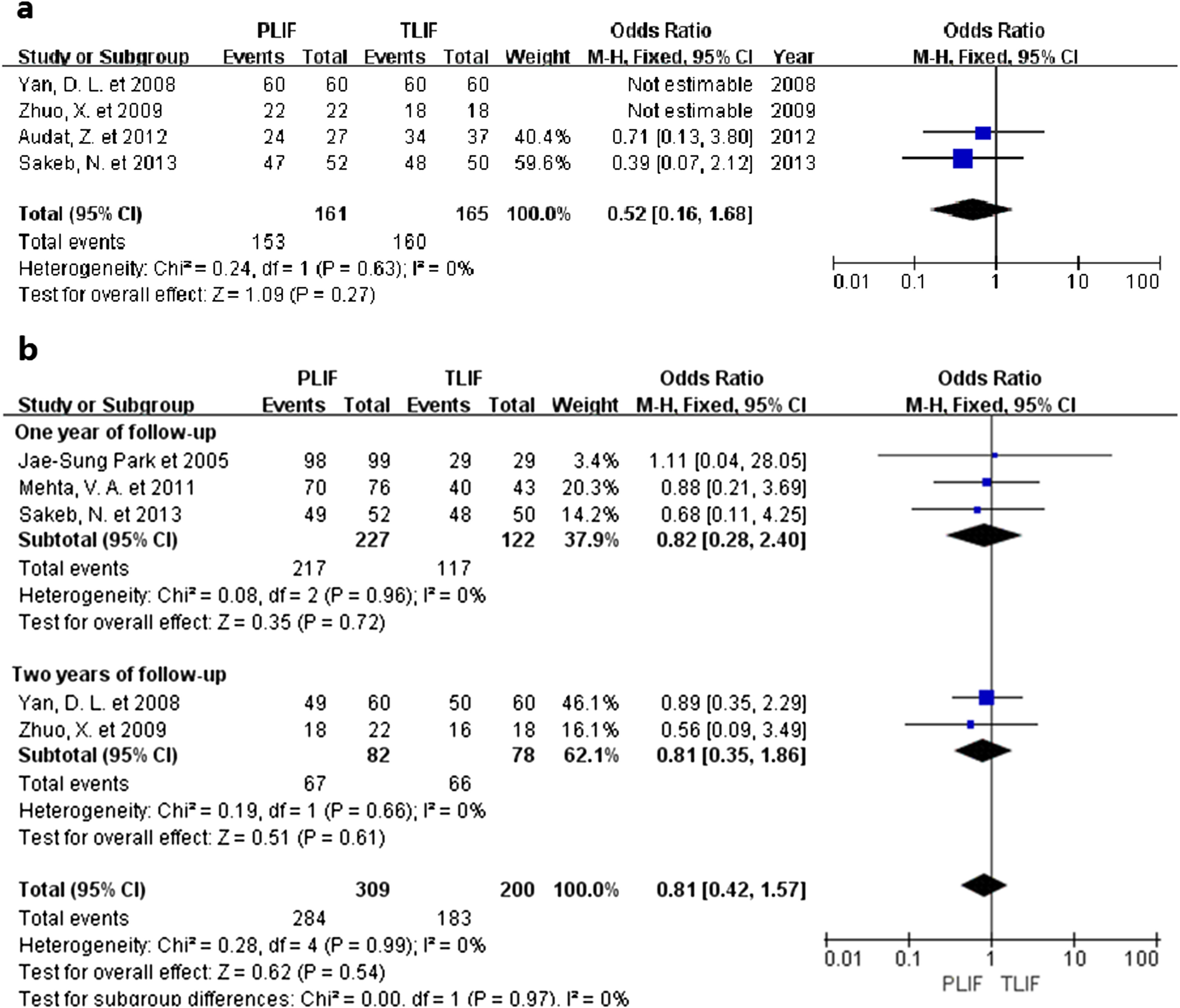

Supplement: Supplementary file 3 — Authors’ original file for figure 3 [file 12891_2014_2303_MOESM3_ESM.tif]

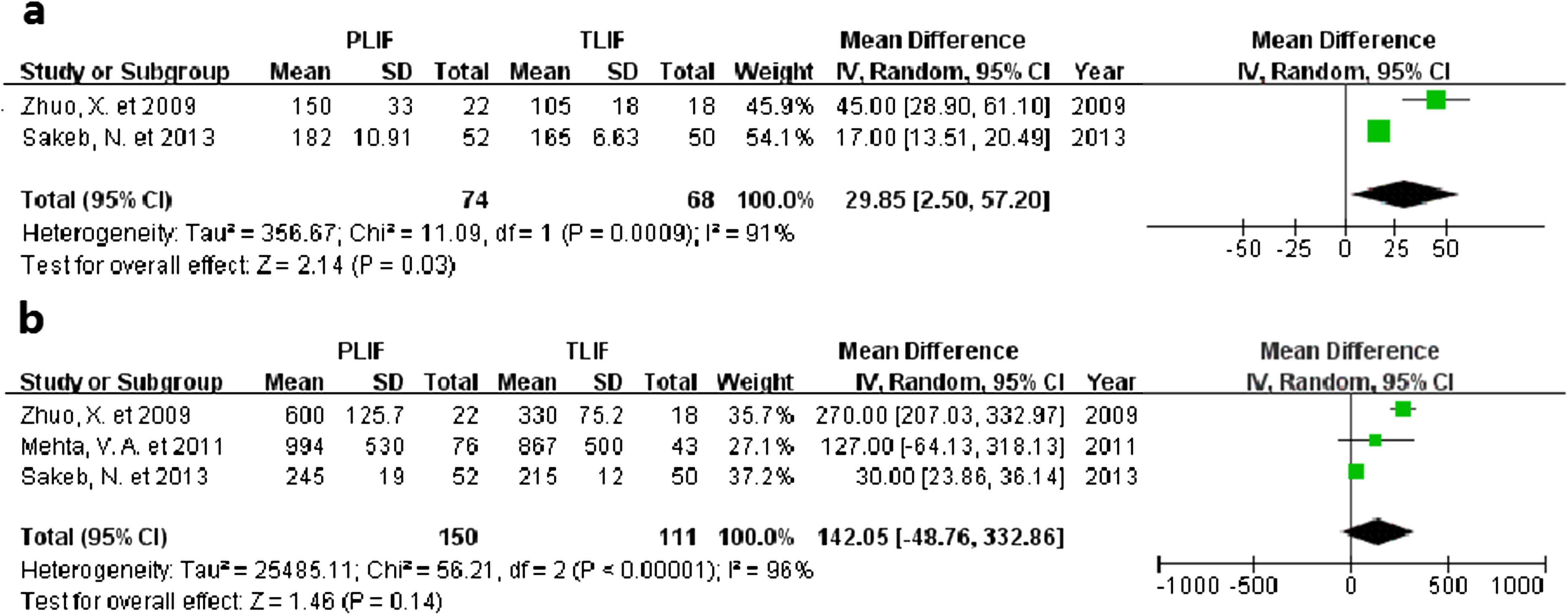

Supplement: Supplementary file 4 — Authors’ original file for figure 4 [file 12891_2014_2303_MOESM4_ESM.tif]
